# The Role of Different Types of Cannabinoids in Periodontal Disease: An Integrative Review

**DOI:** 10.3390/pharmaceutics16070893

**Published:** 2024-07-04

**Authors:** Jaiane Carmelia Monteiro Viana, Gabriela Ellen da Silva Gomes, Francisca Jennifer Duarte Oliveira, Lidya Nara Marques de Araújo, Guilherme Teles, Carlos Fernando Mourão, Bruno César de Vasconcelos Gurgel

**Affiliations:** 1 Department of Dentistry, Federal University of Rio Grande do Norte, Natal 59078-970, Brazil; 2 American Dental Institute, Orlando, FL 32819, USA; 3 Department of Periodontology, Tufts University School of Dental Medicine, Boston, MA 02111, USA

**Keywords:** cannabinoids, periodontal diseases, treatment, inflammation

## Abstract

This integrative review addresses the potential of the Endocannabinoid System (ES) and cannabinoids in the pathogenesis and treatment of periodontal disease (PD). Cannabinoid receptors are expressed in healthy and inflamed periodontal tissues, indicating a potential regulatory role for SEC in oral homeostasis. Healthy periodontal cells express more CB1 receptors, while inflamed sites show increased CB2 receptors. This suggests a dynamic involvement of the SEC in the inflammatory response associated with PD. Cannabinoids such as cannabidiol (CBD) and cannabinoid receptor agonists such as HU-308, anandamide (AEA), and methanamide (Meta-AEA) have demonstrated promising therapeutic potential in studies. CBD has been associated with the control of bone resorption, antibacterial activity, and increased production of gingival fibroblasts, indicating effects in mitigating the progression of PD. HU-308 demonstrated preventive effects against alveolar bone loss, and anti-inflammatory, osteoprotective, and pro-homeostatic properties in animal models of periodontitis. AEA and Meta-AEA have anti-inflammatory effects by reducing pro-inflammatory mediators such as IL-1, IL-6, and TNF-α. The activation of cannabinoid receptors attenuates inflammatory processes, inhibits alveolar bone loss, exerts antibacterial effects, and promotes tissue repair. However, clinical trials are especially needed to validate these results and explore the therapeutic potential of cannabinoids in the treatment of PD in humans.

## 1. Introduction

Periodontal disease (PD) is a chronic inflammatory condition that compromises the teeth’s supporting tissues. This occurs primarily through the immunoinflammatory responses that are triggered by interactions between pathogenic bacteria and host-derived mediators, and contribute to the exacerbation of inflammation and subsequent tissue destruction [1,2]. Some biological systems, i.e., the Endocannabinoid System (ECS), are potential candidates for controlling PD through the modulation of conditions such as pain, inflammation, antimicrobial properties, and tissue repair. This system is a complex signaling network that plays a crucial role in regulating various physiological processes, including immune response, pain modulation, and bone metabolism [3,4]. It is composed of endogenous cannabinoids (endocannabinoids), cannabinoid receptors, and enzymes responsible for the synthesis and degradation of endocannabinoids [5]. They have roles in the cellular activities of osteoblasts, osteocytes, osteoclasts, and gingival fibroblasts which are important for oral homeostasis, as they assist in tissue remodeling [6]. In addition, this system, in isolation or when associated with different types of cannabinoids, has also been purported to hold therapeutic potential for periodontal diseases [6,7].

In general, conventional treatment for PD includes oral hygiene guidance, combined with scaling and root planning that may or may not be associated with adjunctive therapies, such as antimicrobial photodynamic therapy [8,9,10]. It may also require surgical intervention to reduce the depth of periodontal pockets [11]. Furthermore, the local or systemic administration of cannabinoids (substances that bind to Endocannabinoid System (ECS) receptors) has been reported to influence disease control by reducing the expression of inflammatory mediators such as IL-6 and TNF-α [12,13]. CB1 and CB2 receptors are proteins found on types of cells such as B and T lymphocytes, natural killer (NK) cells, monocytes, neutrophils, CD8+ white blood cells, and CD4+ white blood cells. This means that both natural and external cannabinoids have the ability to directly influence how the immune system works by affecting processes like cytokine release, cell growth, and enzyme activation, in effector cells [4].

In this context, cannabinoids comprise a diverse group of molecules that can be endogenous (anandamide (AEA), 2-arachidonylglycerol (2-AG), and Npalmitoylethanolamide (PEA)), synthetic (methanandamide—Meta-AEA- and HU-308) or derived from plants (phytocannabinoids; Δ9-tetrahydrocannabinol (THC), cannabidiol (CBD), cannabichromene (CBC), cannabinol (CBN), among others) [14]. These substances may contribute to the pathogenesis and healing of periodontal tissues, as an increase in the expression of cannabinoid receptors has been shown at diseased periodontal sites, as well as a decrease in inflammatory mediators and greater cell proliferation, following exposure to CBD and AEA, for example [15,16]. Due to their potential immunomodulatory, antibacterial, and regenerative effects, cannabinoids may offer advantages in the treatment of periodontal disease and have been suggested as an adjuvant therapeutic alternative to complement other established therapies [4]. Their effects could potentially augment the benefits already obtained through conventional treatments.

Some studies have investigated the relationship between the ECS, the cannabinoids themselves, and periodontal disease [7,17,18]. Most of these are in vitro studies and preclinical trials that (samples of human tissues/cells or other animals) identify the possible contributions of this system to the progression and/or management of inflamed periodontal tissues, although its role is not clear [3,15,19]. Therefore, the aim of the present study was to evaluate, through an integrative review of in vivo and in vitro studies, the role of ECS in the pathogenesis of PD and to determine the current evidence justifying or recommending the use of cannabinoids in periodontal therapy. 

## 2. Materials and Methods

This integrative literature review aimed to identify the role of ECS in the pathogenesis and therapy of PD in in vitro and in vivo studies. For this purpose, electronic searches were performed in PubMed, Embase, Scopus, Web of Science, and Lilacs databases up to December 2023, using descriptors and terms controlled by MeSH (Medical Subject Headings) and DeCS (Descriptors in Health Sciences), as shown in Table 1.

### 2.1. Eligibility Criteria 

Due to the diversity among the studies, an adaptation of the PICOS strategy was employed to consider the eligibility of studies using P, I, and O:

**P:** Population (bacteria, cells, and other structures inherent in diseased and/or healthy periodontal tissue—from humans, mice, or rabbits)
**I:** Intervention (natural, endogenous, and/or synthetic cannabinoids)
**O:** Outcome (anti-inflammatory, antibacterial, or repair-related effects and prevention of soft and hard tissue damage).

### 2.2. Inclusion Criteria

Only in vitro and in vivo studies that evaluated the role of the ECS in tissues, cells, or microorganisms inherent to the periodontium were considered. Restrictions on language, publication time, and geographical scope were not employed. 

### 2.3. Exclusion Criteria

Descriptive studies, such as literature reviews, case reports, clinical trials, projects/protocols, opinion articles, letters, posters, and conference abstracts were excluded. 

### 2.4. Data Extraction and Analysis

For the selection of studies, the *Rayyan Qatar Computing Research Institute* (QCRI) application was used in two phases. In phase one, duplicate articles were identified, and titles and abstracts were independently screened to exclude studies irrelevant to this review. In phase two, the texts were fully read, applying the previously established inclusion and exclusion criteria. The data collected were as follows: (1) Author and year of publication; (2) Type of study; (3) Intervention; (4) Objective; (5) Cannabinoid dose/administration; (6) Results (main outcomes of the articles related to anti-inflammatory, antibacterial, and tissue repair response); (7) Conclusion.

## 3. Results

### 3.1. Selection of Studies

**Figure 1:**
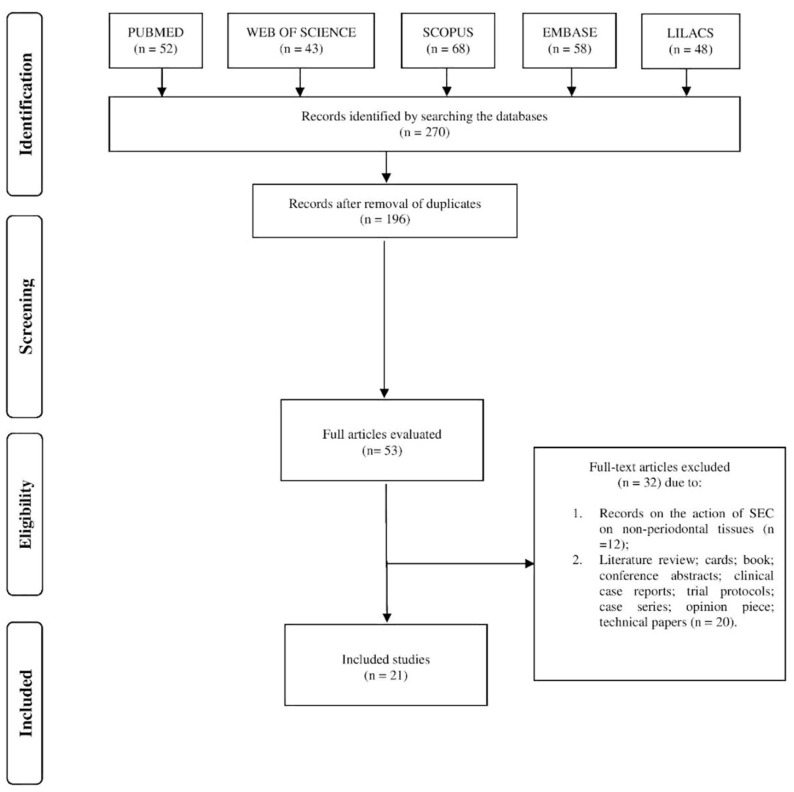
Flow diagram of the selected studies.

After an electronic search in five databases (PubMed, Web of Science, Scopus, Embase, and Lilacs), 270 articles were retrieved, and after the removal of duplicates, 196 remained for us to read the title and abstract. Of these, 21 were included based on the eligibility criteria (Figure 1). 

### 3.2. Characteristics of the Studies

The included studies were published between 2005 and 2022. The majority, eleven (52%), had been published in the last 5 years; three (15%) in the last 10 years, and seven (33%) more than 10 years ago. Regarding the study design, fifteen (71%) articles presented in vitro methodologies; four (19%) in vivo, and two (10%) had both methodologies (Table 1).

With regard to the therapeutic potential of ECS and its ligands in periodontal disease, 10 studies investigated the anti-inflammatory performance of different types and representatives of cannabinoids in structures inherent to the periodontal tissue of humans and mice [3,6,12,15,19,20,21,22,23,24]. Another three studies evaluated their antibacterial activity [21,25,26]; six evaluated the tissue capacity of repair/cell proliferation [15,19,23,27,28,29]; and another six studies investigated the role of these substances in preventing alveolar bone resorption [3,6,13,22,24,30].

**Figure 2:**
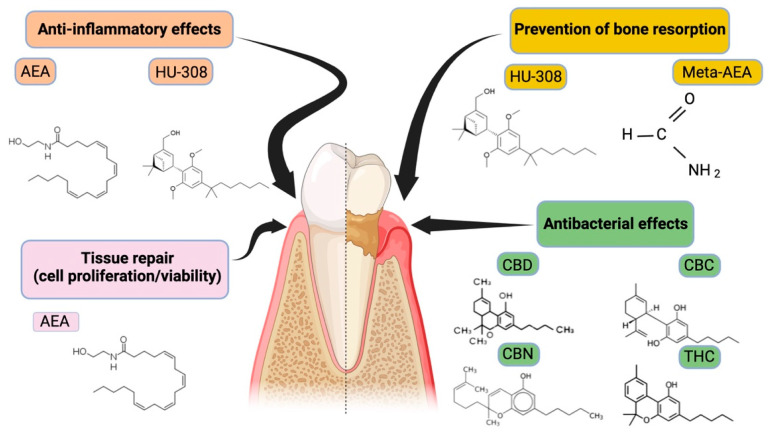
Representation of the main cannabinoid molecules and their respective effects on periodontal disease. Cannabinoids comprise a diverse group of molecules that can be endogenous (anandamide (AEA), synthetic (methanandamide (Meta-AEA), and CB_2_-specific agonist (HU-308)) or derived from plants (phytocannabinoids; Δ9-tetrahydrocannabinol (THC), cannabidiol (CBD), cannabichromene (CBC), cannabinol (CBN)).

**Figure 3:**
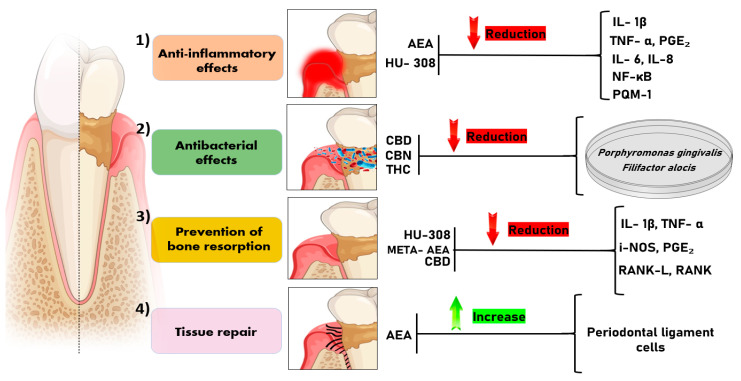
Illustrates the possible mechanisms described in the literature on the effects of cannabinoids and their derivatives on periodontal disease. (1) Anti-inflammatory effect: AEA and HU-308 act to reduce the levels of cytokines involved in the inflammatory cascade (IL-1β, TNF-α, PGE₂, IL-6, IL-8, NF-κB, MCP-1). (2) Antibacterial effect: CBD, CBN, and THC have an inhibitory effect on the growth of periodontopathogenic bacteria, such as *Porphyromonas gingivalis* and *Filifactor alocis*. (3) Prevention of bone resorption: HU-308, META-AEA, and CBD demonstrate the ability to reduce alveolar bone loss by reducing levels of mediators (IL-1β, TNF-α, iNOS, PGE₂, RANKL, RANK) that induce bone resorption. (4) Tissue repair: AEA demonstrates an increase in tissue repair capacity due to its action on the proliferation of periodontal ligament cells. Legend: AEA: anandamide; HU-308: CB2-specific agonist; CBD: Cannabidiol; CBN: Cannabinol; THC: Δ9-Tetrahydrocannabinol; META-AEA: methanandamide; IL-1β: interleukin-1β; TNF-α: tumor necrosis factor alpha; PGE₂: Prostaglandin E2; IL-6: interleukin 6; IL-8: interleukin 8; NF-κB: Nuclear Factor Kappa-light-chain-enhancer of Activated B Cells; MCP-1: Monocyte Chemoattractant Protein-1; iNOS: Inducible Nitric Oxide Synthase; RANK: Receptor Activator of Nuclear Factor Kappa B; RANKL: Receptor Activator of Nuclear Factor Kappa B Ligand.

Regarding the participation of this system in the pathogenesis of periodontal disease, five studies evaluated the expression of CB1 and CB2 receptors in healthy or diseased periodontal tissue samples [16,17,27,31,32]. Considering the types of cannabinoids used in the studies, at least 13 different types were observed. The most studied were the endocannabinoid, anandamide [12,15,20,23,24,27,32,33], the phytocannabinoid CBD [12,15,20,23,24,27,32], and the synthetic cannabinoid, HU-308 [3,12,13]. The chemical structure and effects of these molecules on the periodontium are illustrated in Figure 2. Figure 3 illustrates the possible mechanisms on the effects of cannabinoids and their derivatives on periodontal disease.

### 3.3. Prevention of Bone Resorption by Cannabinoids

Among the six studies that evaluated the prevention of bone resorption, four used synthetic cannabinoids, including HU-308 [3,13,30] and Meta-AEA [6]. HU-308, employed in in vivo studies, significantly reduced alveolar bone loss in animals subjected in parallel to bacterial LPS (4.52 ± 0.19 mm) stimulation, compared with animals receiving LPS alone (5.22 ± 0.14 mm) [13], and similar results were found for Meta-AEA [6]. The only study that investigated the role of a phytocannabinoid concluded that rats subjected to experimental periodontitis and systemic CBD injection showed less bone loss in the furcation region (0.5 mm^2^), compared with those treated with saline (0.9 mm^2^) [22].

### 3.4. Anti-/Pro-Inflammatory Effects of Cannabinoids

Of the studies that investigated the anti-inflammatory efficacy of cannabinoids, seven investigated the performance of synthetic cannabinoids, especially HU-308 [3,12,13,20,30] and Meta-AEA [6,19]. Five studies evaluated the effects of the endocannabinoid AEA [12,15,20,23,24] while two investigated the phytocannabinoids, especially CBD [21,22]. CBD administered in animals subjected to experimental periodontitis [22] and in cells stimulated by LPS from *P. gingivalis* [21] reduced the levels of cytokines such as RANK, RANKL, TNF- α, IL-1β, IL-6, IL-12 p40, and IL-8 [21,22]. The synthetic cannabinoid Meta-AEA, when applied to gingival tissue samples from mouse [6] and human periodontal ligament cells [19] exposed to bacterial LPS, significantly reduced TNF-α, PGE2 [6], IL-6, IL-8, and MCP-1 levels [19]. Similar results were found for HU-308, which demonstrated efficacy mainly in reducing IL-6, TNF-α, and IL-1β in inflamed periodontal tissues [3,12,20]. In contrast, the injection of anandamide in rats with experimental periodontitis significantly reduced TNF-α and IL-1β levels compared with animals treated with saline and the antagonists, AM251 and AM630 [24].

### 3.5. Tissue Repair (Cell Proliferation/Viability) by Cannabinoids

**Figure 4:**
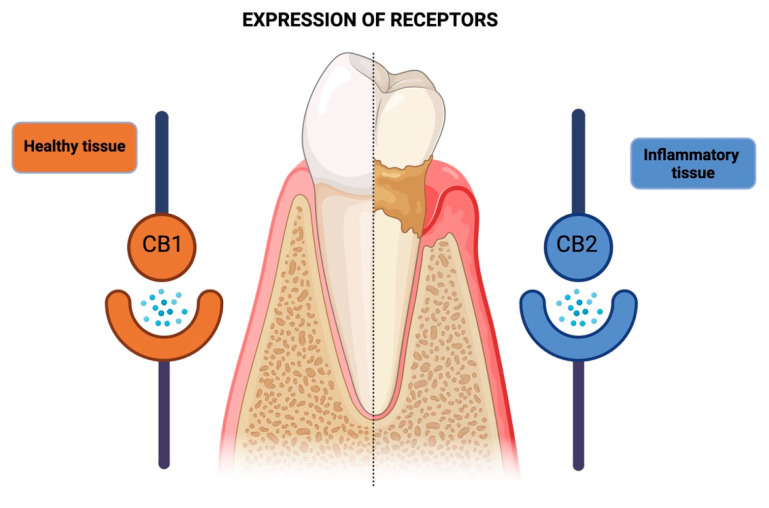
Schematic representation of the differential expression of cannabinoid receptors (CB1 and CB2) in healthy and diseased periodontal tissues. In healthy periodontal tissues, CB1 receptor expression predominates, while inflamed tissues exhibit a higher tendency for CB2 receptor expression. This shift in receptor expression suggests a potential regulatory role of the Endocannabinoid System in the pathogenesis and progression of periodontal disease.

Those studies that analyzed tissue repair capacity (as assessed by cell proliferation or molecule expression) mainly investigated the effects of the endocannabinoid AEA [15,19,27]. In human periodontal ligament cells not exposed to bacterial LPS, anandamide had no significant effect on the proliferation/viability of these cells, while in those submitted to LPS, AEA (10–20 µM) induced a significant increase in proliferation/viability [23]. In addition, the phytocannabinoid, CBD, at low concentrations (0.01–0.05 µM), increased (by 40%) the production of transforming growth factor β (TGF-β), an important cytokine that controls cell proliferation and differentiation [28]. This relationship between receptors and periodontal tissue is illustrated in Figure 4.

### 3.6. Antibacterial Effects of Cannabinoids

With regard to the antibacterial activities of cannabinoids, only the phytocannabinoids have been studied, especially CBD, CBC, CBN, and THC [21,25]. In addition, one study investigated the effect of CBD and CBG when infused in mouthwashes [25]. Exposure of the oral biofilm to the phytocannabinoid, CBD (12.5%), resulted in lower bacterial colony counts in samples taken from patients with gingivitis (mean colony count (MCC) = 5), calculus-associated gingivitis (MCC = 4.9), and from patients with severe PD (MCC = 1.5); these values differed from those of samples from patients with severe periodontitis submitted to Oral B (MCC = 29.8). Meanwhile, CBD (12.5%) administration, especially in patients with periodontitis (CMC = 3.1), also lowered bacterial growth averages, compared with those presented by groups treated with Oral B (CMC = 27.3) or Colgate (CMC = 27.7) [25]. The direct effects of CBD, CBN, and THC on *P. gingivalis* were also investigated. CBD, at concentrations of 5.0 and 10 µg/mL, significantly inhibited bacterial logarithmic growth up to 38 h after exposure, and similar results were observed for CBN and THC [21]. On the other hand, CBD and CBG, when infused in mouthwashes, showed a similar inhibition of bacterial growth to that of 0.2% chlorhexidine, and greater inhibition compared with the inhibition resulting from alcohol-based rinses with essential oils such as thymol, eucalyptol, and menthol (Product A) or fluoride- and potassium nitrate-based products (Product B) [26].

### 3.7. Expression of Receptors (CB1 and CB2)

The expressions of CB1 and CB2 receptors were analyzed to assess the role of the Endocannabinoid System in the pathogenesis of periodontal disease in five studies [21,25]. Compared with CB1, there was a greater tendency towards CB2 expression in healthy [17], inflammatory [16,27,32,33], and healing conditions [31]. Additionally, human gingival fibroblasts exposed to *P. gingivalis* LPS showed a marked expression of CB1 and CB2 messenger RNAs [32]. However, in healthy cells and tissues, CB1 levels were higher compared with CB2 [31,32] although the expression of this receptor was lower or absent in some studies [17,27].

The materials and data for this study are openly available on the Open Science Framework (OSF): https://doi.org/10.17605/OSF.IO/GFDZ2.

The studies summaries are included in Table 2.

## 4. Discussion

The pathogenesis and therapeutics of PD are still being studied, with the aim of making its prevention and management more efficient. Currently, there are a variety of cannabinoid-based oral products on the market that promise analgesic, anti-inflammatory, and antibacterial efficacy; such products include chewing gums, dentifrices, oils, capsules, sprays, and mouthwashes [34]. However, no evidence or clinical studies are available that directly involve the use of these products for the prevention or treatment of this condition. Thus, this integrative review of preclinical studies investigated the role of the ECS and different types of cannabinoids in the pathogenesis and therapy of periodontal disease. 

Since 2006, in vitro and in vivo studies have been investigating the performance of natural, synthetic, and endogenous cannabinoids, with regard to their anti-inflammatory, antibacterial properties, participation in tissue repair, and prevention of alveolar bone loss, in relation to periodontal disease [13,15,16,21,32]. Through this review, the role of cannabinoids in the prevention of alveolar bone resorption and the modulation of inflammatory responses, tissue repair, and antimicrobial effects was synthesized. The authors identified the potential of endogenous, synthetic, and natural cannabinoids in reducing inflammatory processes, exhibiting antibacterial activity, reducing alveolar bone loss, and promoting tissue repair in both in vitro and in vivo studies. However, none of the included studies directly investigated the analgesic potential of different types of cannabinoids on periodontal tissues. This may be justified by the profile of the studies (mostly in vitro), but is also due to the characteristics of periodontal disease, as the patient usually does not manifest pain, except in advanced and/or acute stages [35].

Proportionally, most studies have investigated the anti-inflammatory capacity of synthetic and endogenous cannabinoids and, to a lesser extent, phytocannabinoids. The findings reveal that the exposure of rat or human periodontal tissue/cells to these substances significantly reduces the expressions of cytokines relevant to periodontal destruction, such as TNF-α and IL-1β [3,12,20,24]. The importance of these findings is supported by evidence, for example, that TNF-α participates in alveolar bone resorption through different pathways, such as osteoclast differentiation and maturation [36]. IL-1β increases the expression of collagenolytic enzymes and matrix metalloproteinases (MMPs), contributing to the degradation of the extracellular matrix and, in turn, leading to bone resorption and tissue destruction [37]. As such, these anti-inflammatory findings align with the observed decrease in alveolar bone loss observed in animals that underwent cannabinoid treatment for experimental periodontitis [6,13,22].

With regard to the healing, repair, and regeneration of periodontal tissues, the effects of endogenous and synthetic cannabinoids on the proliferation and cell viability of human gingival fibroblasts and periodontal ligament cells [15,23,27] were mainly investigated. This may represent an important indicator, as gingival fibroblasts participate in tissue repair and remodeling, after procedures such as scaling and root planning and periodontal surgery. Furthermore, in general, periodontal ligament cells constitute the supporting tissue of the teeth in the alveolus, participate in the sensory function due to their abundant innervation, contribute to the dissemination of occlusal forces to the supporting bone, and favor cell formation and nutrition of bone, cementum, and gingiva [27].

The role of CBD in the therapy of several diseases (rheumatoid arthritis, multiple sclerosis, Alzheimer’s disease, anxiety disorders) is consolidated in the medical literature due to its analgesic, antitumor, anti-inflammatory, and central nervous system effects, among others [38]. However, only 5 of the 21 studies included in this review studied this phytocannabinoid with regard to its effects on the control of bone resorption and repair [16,22], antibacterial activity [21,25,26], and cell proliferation [28]. Traditionally, the main objective when studying the management of PD is to reduce the microbial load, especially periodontopathogens. Phytocannabinoids (CBD and THC) have already shown efficacy against gram-positive microorganisms such as *Staphylococcus aureus* [39] and, similarly, CBD reduced the growth of some gram-positive and negative microorganisms (*P. gingivalis* and *Filifactor alocis*) [19]. In addition, phytocannabinoids (isolated or infused in mouthwashes) also demonstrated antimicrobial activity that was superior or equal to that of traditional oral products such as 0.2% chlorhexidine and dentifrices, suggesting that these substances hold antibacterial potential [25,26]. 

With regard to the expressions of CB1 and CB2, there was no consensus among the studies, and differences were observed in the expressions of these receptors according to the characteristics of periodontal tissues [17,31]. This may be related to the types of stimuli examined, such as experimental periodontitis [27] and mechanical stress [31] or due to differences in cells and tissues, as some studies evaluated gingival tissue from rats [17,27] and humans [31], as well as gingival fibroblasts [16,32] and periodontal ligament cells from humans [31]. 

Despite the potential role of cannabinoids in preventing alveolar bone resorption, modulating inflammatory responses, promoting tissue repair, and exerting antimicrobial effects, some disadvantages should be considered. The long-term effects of cannabinoids on periodontal tissues remain unknown, and predicting their effectiveness and appropriate dosage is challenging due to the close relationship between the actions of these substances and the individual’s ECS and immune system. Moreover, the lack of standardization in dosing inherent to cannabinoid-based products may lead to inconsistent therapeutic outcomes [4].

The clinical relevance of these findings suggests potential improvements in periodontal clinical parameters, which may prevent, reduce, or limit the destruction of periodontal tissues inherent in the immune-inflammatory response of periodontal disease. However, although in vitro and in vivo studies provide contributions to the development of clinical trials, the limitations of these trials include the irreproducibility of the interaction between the products and substances tested with the human host, since the tests are restricted to inoculation in groups of cells isolated from humans or other animals and to cultivation in tubes or glass plates, for example. Finally, other limitations exist, such as the lack of standardization of studies, techniques, and analyses, which could provide greater data replicability, as well as the fact that laboratory conditions do not always occur simultaneously with real conditions. Further randomized and controlled clinical studies are needed to confirm the safety, tolerability, toxicity, efficacy, and optimal dosages of these compounds.

## 5. Conclusions

Preclinical studies evidence the positive role of the Endocannabinoid System (ECS) and different types of cannabinoids—such as endogenous (anandamide (AEA)), synthetic (methanandamide (Meta-AEA), and HU-308), and natural (Δ9-tetrahydrocannabinol (THC), cannabidiol (CBD))—in the pathogenesis and therapeutics of conditions affecting oral tissues and cells. Notable contributions of this system and its ligands include the potential for preventing bone resorption, anti-inflammatory effects, tissue repair capabilities, and antimicrobial effects. However, future applications must consider limitations, such as the safety of natural, synthetic, and/or endogenous cannabinoids and their products, optimal dosages, and interactions between products, which need to be verified for the treatment of periodontal disease.

## Figures and Tables

**Table 1:** Search strategy.

PubMed, Web of Science, and Embase	Lilacs and Scopus
(Periodontics OR “Periodontal Diseases” OR “Periodontal Ligament” OR Periodontitis OR “Chronic Periodontitis” OR “Chronic Periodontitides” OR gingivitis) **AND** (Cannabinoids OR CBD OR “cannabigerolic acid” OR cannabinol OR cannabigerol OR cannabidiol OR cannabichromene OR Cannabite OR phytocannabinoid OR HU 308 OR anandamide (AEA) OR “β-Caryophyllene” OR methanandamide OR “Cannabinoid Receptor Agonists” OR “Agonist, Cannabinoid” OR “Cannabinoid Receptor” OR “Cannabinoid Receptor CB1” OR “Receptor, Cannabinoid, CB1” OR “Cannabinoid Receptor CB2” OR “Receptor, Cannabinoid, CB2” OR Endocannabinoid OR CB1 OR CB2 OR THC) **AND** (“Alveolar Bone Loss” OR “bone resorption” OR “bone healing” OR “gingival inflammation” OR Gingivitis OR Inflammation OR “Anti-Inflammatory Agents” OR “Anti Inflammatories” OR anti-inflammatories OR “Anti-Bacterial Agents” OR “Agent, Anti-Bacterial” OR Antibiotic OR Bactericide OR “Cell Proliferation” OR “Cellular Proliferation” OR “Cell Number Growth”)	(“Periodontal Diseases” OR “Periodontal Ligament”) **AND** (Cannabinol OR “Cannabinoid Receptor” OR Endocannabinoid) **AND** “Alveolar Bone Loss” OR Anti-inflammatories OR Bactericide OR “Cellular Proliferation”

**Table 2:** Summary of included studies.

Author, Year, Type of Study	Intervention, Dose- Administration	Objective	Methodology	Results	Conclusion
QIAN, 2010 [30] In vitro	**Test:** HU-308 **Control:** untreated CLPH	To investigate CB2 expression and the effect of its activation on osteogenic differentiation of CLPH.	CLPH were treated with HU-308 and the RT-PCR technique was used to identify the expression of RCB2 and the mRNA expressions of osteogenic and osteoclastogenic genes.	**Bone resorption:** The mRNA expression of osteogenic genes (Runx2 and OPN) increased significantly (*p* < 0.05) from 24 h of HU308 treatment, and increased over time. This was also observed for SPO, OC, FA, and COL I mRNA. Regarding the effect of HU-308 on osteoclastogenic genes, a significant increase in OPG mRNA expression was observed after 48 h, but a significant reduction in RANKL mRNA expression was seen after 24 h (*p* < 0.05).	Activation of CB2 through binding to HU-308 can enhance osteogenic differentiation in CLPH, in addition to contributing to alveolar bone metabolism through bone remodeling.
OSSOLA, 2020 [13] In vivo	**Test**: Rats with EP + HU 308 (500 ng/mL, 200 µL injectable per tooth) **Control**: Healthy mice Untreated EP (LPS) rats	To evaluate the effects of HU 308 treatment on the oral health of rats subjected to early periodontitis.	Eighteen rats (six rats per group): (1) control; (2) rats with EP; and (3) rats with EP treated daily with HU 308 were evaluated for POA	**Bone resorption:** POA in maxillary and mandibular molars was significantly lower for HU 308-treated rats (2.50 mm; 4.52 mm), compared with untreated (3.02 mm and 5.22 mm) (*p* < 0.05). In healthy rats, POA in mandibular teeth was lower (*p* < 0.01) than that of the other groups.	HU 308 demonstrated a preventive effect in the treatment of POA in rats with EP.
NAPIMOGA, 2009 [22] In vivo	**Group 1:** Rats without EP + Saline Solution **Group 2:** Rats with EP + Saline Solution **Group 3:** Rats with EP + CBD (5 mg/kg, injectable for 30 days)	To evaluate the effect of systemic injection of CBD in rats submitted to EP.	CBD dissolved in 2% Tween 80 and saline solution. Solutions were prepared at a volume of 1 mL/kg for 30 days.	**Bone resorption:** CBD (5 mg/kg) significantly (*p* < 0.05) inhibited the volume of bone loss in the bifurcation region. **Anti/Pro-inflammatory effect:** CBD exhibited lower expression levels of RANK, RANK L, TNF-α, and IL-1β (*p* < 0.05), compared with animals that received saline as treatment.	CBD may control bone resorption and inflammatory response during the progression of experimental periodontitis in rats.
OSSOLA, 2012 [6] In vitro In vivo	In vitro **Group 1:** Control **Group 2:** LPS **Group 3:** LPS + Meta-AEA **Group 4:** Meta-AEA (500 ng/mL) In vivo: **Group 1:** Control **Group 2:** Serum injection **Group 3:** Rats with EP (LPS) **Group 4:** Rats with EP (LPS) + Meta-AEA—(500 ng/mL)	To evaluate the effect of treatment with the synthetic cannabinoid, Meta-AEA, on the progression of periodontitis in rats, with regard to anti-inflammatory activity and prevention of alveolar bone loss.	In vitro: gingiva from healthy rats were dissected and distributed into the 4 study groups In vivo: rats were divided and daily topical application of serum or Meta-AEA was performed at the LPS injection sites, during the 6 weeks of each experiment.	**Bone resorption** In vivo: EP + Meta-AEA animals compared with untreated EP animals showed significantly lower POA (*p* < 0.05) and lower expression of iNOS (*p* < 0.01) and TNF-α (*p* < 0.05). **Anti/Pro-inflammatory** In vitro: Meta-AEA + LPS significantly (*p* < 0.01) reduced TNF-α and PGE2 levels, compared with the LPS group. TNF-α levels were significantly higher for LPS (*p* < 0.001) and LPS + Meta-AEA (*p* < 0.01), compared with control.	The use of Meta-AEA, in vivo and in vitro, reduced the levels of proinflammatory cytokines, especially TNF-α. In vivo use significantly decreased alveolar bone loss and the amount of iNOS.
OSSOLA, 2016 [3] In vivo	**Group 1:** Control **Group 2:** 1% ethanol in saline solution **Group 3:** Rats with EP (LPS) + 1% ethanol in saline **Group 4:** Rats with EP (LPS) + HU-308—(500 ng/mL, 200 µL injectable per tooth)	To evaluate the anti-inflammatory, osteoprotective, and pro-homeostatic effects of HU308 treatment on the oral health of rats submitted to EP.	The animals were divided into the 4 groups and treated according to each type of intervention, during the 6 weeks of each experiment.	**Bone resorption:** Animals treated with HU 308 had lower POA in the buccal maxilla (1.4 mm vs. 1.8 mm *p* < 0.05) and lingual mandible (3.5 mm vs. 4.9 mm, *p* < 0.05), as well as higher inter root bone percentage (50% vs. 39%) (*p* < 0.01), when compared with the diseased rats that received saline. **Anti/Pro-inflammatory effect:** HU-308 treatment resulted in lower levels of iNOS, TNF-α, and PGE2, compared with the LPS group (*p* < 0.05).	HU-308 may exhibit anti-inflammatory, osteoprotective, and pro-homeostatic effects in oral tissues of rats with LPS- induced periodontitis.
ABIDI, 2020 [12] In vitro	**Group**: FLPHs Control **Group**: FLPHs + HU 308 (14 µM) **Group**: FLPHs + AEA (16 µM) **Group**: FLPHs + IL1β + AEA **Group**: FLPHs + IL1β + HU 308	To understand the role of CB2 during periodontal inflammation and identify anti-inflammatory agents for drug development to treat periodontitis.	AEA and HU-308 were tested for effects on cytokines, chemokines, and angiogenic and vascular markers expressed by FLPHs, which were divided into groups according to the type of exposure: Control; AEA or HU 308; IL-1β + AEA or IL-1β + HU 308.	**Anti/Pro-inflammatory effect:** In FLPHs, AEA alone increased the expression of IFN-γ and IL-13, but this was not significant. In the AEA + IL-1β group, IFN-γ (*p* < 0.01), IL-6 (*p* < 0.0001), TNF-α (*p* < 0.05) were significantly increased, relative to IL-1β alone. In contrast, HU 308 treatment slightly increased IL-13 levels, while for HU 308 + IL-1β, compared with IL-1β, there were significant reductions for all other cytokines; IFN-γ, IL-1β, IL-2, IL6, TNF-α, FEC-GM.	AEA exhibited both pro- and anti-inflammatory properties in FLPHs, whereas HU 308 demonstrated only anti-inflammatory activity.
RETTORI, 2012 [24] In vitro	**Test**: AEA and/or CB1 antagonists (AM 251) and CB2 (AM 630) **Control:** Experimental Periodontitis + Stress + sterile saline solution (30 µL, injectable 2 times daily for 7 days)	To investigate the anti-inflammatory role of AEA in experimental periodontitis in rats.	Animals with EP and stressed received different treatment and were investigated for inflammatory response.	**Anti/Pro-inflammatory effect: ** EP rats receiving only AEA had lower corticosterone levels (40 ng/mL) than the control group (60 ng/mL, *p* < 0.05). Similarly, TNF-α levels in the gingiva were lower in the AEA group (30 pg/mg) compared with the control group (45 pg/mg, *p* < 0.001) and the antagonist + AEA group (50 pg/mg *p* < 0.05). IL-1β levels in animals with EP were much lower in those receiving AEA (DOR% = 100), whereas antagonists alone had no effect (DOR% > 150).	The endocannabinoid AEA may decrease the inflammatory response in periodontitis even during stress.
NAKAJIMA, 2006 [32] In vitro	AEA (1, 5, and 10 µM)	To assess the levels of AEA in HRGC and CB1 and CB2 receptor expressions in gingival fibroblasts, and to identify the role of AEA in periodontitis.	Levels of AEA in the HGF and of CB1 and CB2 in gingival tissues or HGFs were assessed.	**Anti/ pro-inflammatory effect: ** Amount of AEA in diseased FGH was 16.4 ± 9.30 ng and dose-dependently reduced LPS-induced IL-6, IL-8, PQM-1, and *NF-K* β production in FGH. While CB1 was expressed in all types of HGF, but especially in those from diseased patients, whereas CB2 was barely detected in health and highly expressed in disease.	AEA and its receptors, CB1 and CB2, are present in periodontal tissues and may regulate cellular pathways leading to the inflammatory response.
OZDEMIR, 2014 [23] In vitro	**Test:** AEA and 2 AG (0.1 to 20 µM) **Control:** Cells stimulated with DMEM + 1% SFB.	To determine the impact of AEA and 2-AG on CLPH response to *P. gingivalis* LPS exposure.	CLPH were cultured in DMEM (in the presence or absence of LPS) and further subjected to AEA and 2-AG.	**Anti/pro-inflammatory effect:** Treatment of these cells with 10 µM of 2-AG resulted in significant (*p* < 0.05) increase in IL-6 gene expression level compared with 1 µM concentration. While 10 µM of AEA induced a significant decrease (*p* < 0.05) in the gene expression levels of IL-6, IL-8, and PQM-1 stimulated by LPS compared with those of cells that did not receive AEA. **Cell** proliferation/viability: Only AEA demonstrated a significant increase (*p* < 0.01) in proliferation/viability of CLPHs in the presence of LPS, compared with the same group receiving lower concentrations of the substance.	AEA and 2-AG may play an important role in modulating periodontal inflammation.
ZHANG, 2020 [19] In vitro	**Group 1:** CLPH + Meta-AEA (0.03, 0.1, 0.3, 1, 3, 10, or 30 μM **Group 2**: CLPH + Meta-AEA (0.03, 0.1, 0.3, 1, 3, 10, or 30 μM) + LPS **Group 3**: untreated CLPH	To investigate the effect of Meta-AEA on the production of pro-inflammatory mediators in CLPH.	CLPH were cultured in DMEM (in the presence, or absence, of Meta-AEA) and some groups were also subjected to *P. gingivalis* LPS exposure.	**Anti-/Pro-inflammatory effect: ** There was no significant effect of Meta-AEA (up to 10 μM) on gene expression levels and IL-6, IL-8, and PQM-1 protein production in healthy cells. In contrast, in CLPH exposed to bacterial LPS, gene expression and the protein production of IL-6, IL-8, and PQM-1 decreased significantly (*p* < 0.05) with 10 μM Meta-AEA. **Cell proliferation/viability:** Meta-AEA (up to 10 μM) exerted no significant effect, but significantly inhibited at a concentration of 30 μM, compared with the control.	Inflammatory response in CLPH may be influenced by ECS activation, since at certain concentrations, Meta-AEA may contribute to decrease interleukins.
RAWAL, 2012 [28] In vitro	**Test:** CBD (0.01–30 µM) **Control:** (DMEM + gentamicin + 1% MeOH)	To determine the effects of CBD on the fibrogenic and matrix degradation activities of human gingival fibroblasts.	Human fibroblasts were incubated in appropriate culture medium and exposed to CBD diluted in MeOH (1%).	**Cell proliferation/viability:** Compared with the control group: CBD (0.01–0.05 µM) increased the production of FTC-β by 40%, and CBD (2 µM) significantly decreased the production of MMP-1 (*p* < 0.05) and MMP-2 (*p* < 0.001). In addition, CBD ≥ 0.5 µM significantly increased fibronectin levels.	Low levels of CBD may contribute to fibrotic gingival enlargement by increasing the production of TGF-β and fibronectin in gingival fibroblasts, and high levels of this substance decrease the production and activity of MMP.
JÄGER, 2020 [15] In vitro	**Test:** AEA and PEA (50 µM) **Control:** untreated CLPH	To evaluate the impact of endocannabinoids AEA and PEA on the expression of inflammatory molecules in CLPH at rest and under simulated mechanical loading.	CLPH were cultured in DMEM and the test group was subjected to CII + exposure, or not, to AEA or PEA. Untreated samples served as control.	**Anti/Pro-inflammatory effect:** In CLPH, the centrifugation-induced inflammation (CII) + PEA group resulted in a significant increase in the expressions of inflammatory cytokines (IL-1β; IL-6; TNF-α) and of CB1 and CB2, compared with the control. CII + PEA reduced the expressions of these cytokines and of CB1 and CB2, compared with the control (*p* < 0.05), CII and CII + PEA. Regarding CLPH proliferation, AEA was able to partially inhibit the cell reduction caused by CII (*p* < 0.05, after 10 h, compared with CII), while PEA did not contribute to cell proliferation.	AEA revealed anti-inflammatory properties and contributed to preservation of cellular integrity. While PEA exacerbated the proinflammatory effects.
ABIDI, 2018 [20] In vitro	**Test 1:** AEA (16 µM) **Test 2:** HU-308 (7.3 µM) **Test 3:** SMM-189 (13 µM) **Control:** DMSO + Ethanol + dH2O	To evaluate the anti-inflammatory efficacy of AEA and HU 308 on IL-6 and PQM-1 in FLPH.	FLPH cultured in appropriate medium and exposed to LPS from *P. gingivalis* + TNF-α and/or IL-1β. In addition, these cells were exposed to HU-308, AEA, or SMM-189, while the control group was subjected to DMSO + alcohol + dH2O.	**Anti/Pro-inflammatory effect:** AEA alone increased IL-6 levels but significantly decreased in the groups exposed to AEA + TNF-α (*p* < 0.05) and AEA + IL-1β (*p* < 0.01), compared with TNF- α and IL-1β alone. Similarly, IL-6 levels were reduced in HU-308+LPS (*p* < 0.001); HU-308 + TNF-α (*p* < 0.05); and HU-308 + IL-1β (*p* < 0.0001) groups, compared with cells exposed to LPS; TNF-α; or IL-1β, respectively. Both AEA and HU308 reduced PQM-1 levels.	AEA and HU 308 had anti-inflammatory effects in PD, as they reduced IL-6 levels and PQM-1 in FLPH. But AEA may also exhibit pro-inflammatory responses.
GU, 2019 [21] In vitro	**Test**: CBD, CBN, and THC (0.0–10.0 µg/mL) **Control**: Samples not exposed to phytocannabinoids	To examine the influence of the main subtypes of marijuana-derived phytocannabinoids in association with oral pathogens: *P. gingivalis*, *Filifactor allocis*, and *Treponema denticola*.	Bacteria were grown in appropriate culture medium, in the presence or absence of phytoncabinoids.	**Antibacterial effect:** CBD, CBN, and THC (5–10 µg/mL) suppressed the growth of *P. gingivalis* and *F. allocis*, but not *T. denticola.* **Anti/Pro-inflammatory effect:** CBD suppressed the release of TNF-α, IL-6, IL-12 p40, and IL-8 from monocytes stimulated by LPS or by one of the 3 bacterial types.	Marijuana-derived phytocannabinoids can suppress bacterial growth and the release of pro-inflammatory cytokines.
STAHL, 2020 [25] In vitro	**Test:** CBD, CBC, CBN, CBG, (12.5%—Direct contact) **Control:** ACBG; Oral B, Colgate, Cannabite F	To compare the effectiveness of commercial oral hygiene products and cannabinoids in reducing the bacterial content of dental biofilm of patients with periodontal health and disease.	Dental biofilm from participants was collected and spread directly into Petri dishes, previously divided into 4 quadrants treated with cannabinoids or toothpaste (undiluted).	**Antibacterial effect:** The highest CMC occurred in the Oral B (29.8) and Colgate (30.6) treatments vs. CBG (3.5) and CBC (3.8), in the periodontitis patient samples. Overall, the CMC in the cannabinoid treatments was significantly lower than that recorded in any of the toothpastes tested.	Cannabinoids have the potential to be used as an effective antibacterial agent against bacteria associated with dental biofilm.
VASUDEVAN, 2020 [26] In vitro	**Test**: CBD-MW; CBG-MW **Control**: Product A (essential oils and alcohol) Product B (No fluoride alcohol) **Positive control:** 0.2% Chlorhexidine (30 µL wells and 15 µL disk-diffusion)	To evaluate the bactericidal activity of cannabinoid-infused mouthwashes against dental biofilm bacteria from patients with gingivitis and periodontitis.	An aliquot of biofilm collected from patients with PD was spread on plates containing wells or disk diffusion with rinses. After incubation, the diameter of the zone of inhibition was measured manually.	**Antibacterial effect:** The mean zones of inhibition of samples from all patients for CBD-MW (18.1 mm) and CBG-MW (17.7 mm) were similar to that of the 0.2% chlorhexidine group (16.8 mm). The CIM revealed bacterial growth from the 3rd dilution (Product A and B); 8th dilution (CBDMW and CBG-MW); and 10th dilution (0.2% chlorhexidine).	CBD-MW and CBG-MW showed similar or better bactericidal efficiency than the positive control (0.2% chlorhexidine).
KOZONO, 2010 [27] In vitro	**Test:** AEA and 2-AG	To elucidate the role of ECS in periodontal healing.	FCG was collected from patients undergoing periodontal surgery. In animals, EP was induced to assess CB1 and CB2 expression. HGFs were cultured and exposed, or not, to CB1 and CB2 antagonists and agonists (AEA).	**Cell proliferation/viability:** In the GFR of patients undergoing surgery, AEA levels were significantly increased after 3 days (*p* < 0.05), compared with pre-surgery, but 2-AG levels were virtually unchanged. AEA via CB1 and CB2 significantly increased (*p* < 0.05) the proliferation of HGFs, compared with the control. **Expression of receptors:** Upon EP, CB1 and CB2 were expressed in scar tissue cells, but in healthy tissue expression, expressions were low or absent.	ECS may have an important modulatory role in periodontal healing, through proliferation of gingival fibroblasts via upregulation of CB receptor expression and increased AEA levels at wound sites.
LIU, 2019 [17] In vitro	**Test:** 1 µM THC **Control:** HGFs not exposed to THC	To investigate the distribution of RCBs in periodontal tissues as well as the effects of HCT on cell adhesion and migration of periodontal fibroblasts to investigate its role in tissue regeneration and healing.	Localization of CB1 and CB2 in gingival tissue collected from rats was performed by immunohistochemistry. HGFs were also cultured in plates containing 6 wells, in which culture media with or without THC were deposited.	**Cell proliferation/viability:** Regarding the HGFs exposed to THC, a significant increase (*p* < 0.05) in the migration of these cells towards this cannabionoid was observed, compared with the control group, after 3 h. **Receptor expression: ** CB1 was decreased in periodontal tissues, although its expression was higher in perivascular and immune cells (macrophages, lymphocytes, and dendritic cells). CB2 was identified at high levels in tissues, especially in the EJ, LP, TC, OA.	ECS may be a therapeutic target to treat periodontal diseases and induce periodontal regeneration.
NAVARRO-SAIZ, 2022 [16] In vitro	**Test:** 10 µM CBD **Control:** SH-SY5Y and monocyte-derived macrophages	To define the presence and activity of cannabinoid receptors in HGFs.	HGFs were cultured in appropriate culture medium and exposed or not to bacterial LPS.	**Expression of receptors:** CB1 and CB2 were detected in HGFs, especially upon exposure to inflammatory agents (LPS and POLY I:C). In addition, exposure of these cells to CBD resulted in a significant increase (*p* < 0.001) in intracellular calcium concentration, compared with unstimulated cells.	FGHs express higher amounts of CB1 and CB2 upon inflammatory stimuli and CBD may influence a higher calcium influx into these cells, which demonstrates its participation in cell biological activity.
KONERMAN, 2017 [31] In vivo In vitro	NA	To analyze the expressions of CB1 and CB2 in periodontal tissues, under physiological and inflammatory conditions.	Immunohistochemical staining for CB1 and CB2 was performed on biopsies of human and rat periodontal tissues, both diseased and healthy. Gene expressions of cannabinoid receptors in CLPH were investigated by quantitative methods	**Expression of receptors:** In healthy periodontal tissues, CB1 was significantly more expressed (13.5% ± 1.3 of total tissue fraction) compared with CB2 (7.1% ± 0.9) in LPH structures. In diseased LPH structures, CB1 expression decreased, while CB2 increased but without significant difference.	CB1 and CB2 are expressed in periodontal tissues with distinct expression changes in different inflammatory conditions.
YAN, 2019 [33] In vitro	**Test 1:** Meta-AEA (10 µM) **Test 2:** AM251	To identify the role and mechanisms of CB1 in osteogenic differentiation of LP stem cells in an inflammatory environment.	CTLPH were stimulated by TNF-α and IFN-γ. In addition, the selective CB1 agonist (Meta-AEA) and its selective antagonist (AM251) were used.	**Expression of receptors:** CB1 blockade (via the antagonist AM251) inhibited in vitro CTLPH mineralization, compared with the control group (*p* < 0.05). Over the weeks, CB1 inhibition contributed to the reduction of bone sialoprotein. Meta-AEA, by binding to CB1, promoted greater in vitro mineralization of CTLPH (*p* < 0.01), compared with the untreated group.	CB1 can activate the osteogenic differentiation potential of CTLPH, under inflammatory conditions and Meta-AEA exposure.

Source: prepared by the author (2023). Legend: **CLPH:** human periodontal ligament cells; **CB2:** cannabinoid receptor type 2; **RT-PCR**: reverse transcriptase reaction followed by polymerase chain reaction; **mRNA:** messenger ribonucleic acid; **Runx2:** RUNX family transcription factor 2; **OPN**: osteopontin; BSP: bone sialoprotein; **OC**: osteocalcin; **FA:** alkaline phosphatase; **COL I:** collagen type I; **OPG**: osteoprotegerin; **RANK**: receptor activating nuclear factor Kappa β; **RANK L**: receptor activating nuclear factor kappa β ligand; **EP:** experimental periodontitis; **LPS**: lipopolysaccharide; **POA**: alveolar bone loss; **CBD**: cannabidiol; **TNF-α**: tumor necrosis factor alpha; **IL-1β**: interleukin-1β; **MetaAEA**: methanandamide; **PGE-2:** Prostaglandin E-2; **iNOS:** Nitric Oxide Synthase; **AEA**: anandamide; **FLPHs**: human periodontal ligament fibroblasts; **IFN-γ**: interferon-gamma; **IL-13:** interleukin-13; **IL-2:** interleukin-2; **IL-6**: interleukin-6; **G-CSF:** granulocyte-macrophage colony-stimulating factor; **CB1:** cannabinoid receptor type 1; DOR%; **G-CSF**: human gingival crevicular fluid; **IL-8:** interleukin-8; **PQM-1**: monocyte chemoattractant protein; **NF-KB**: nuclear factor kappa-light-chain-enhancer of activated B cells; **2-AG:** 2-arachidonoylglycerol; **DMEM**: Dulbecco’s modified Eagle’s medium; **SFB**: fetal bovine serum; **CLPH:** human periodontal ligament cells; **ECS:** Endocannabinoid System; **MeOH:** methanol; **TCF-β:** transforming growth factor β:; **MMPs-1 and 2**: matrix metalloproteinases 1 and 2; **PEA**: palmitoylethanolamide; **CII**: centrifugation-induced inflammation; **SMM-189**: it is a potent and selective CB2 inverse agonist; **DMSO**: Dimethyl Sulfoxide; **PQM1:** monocyte chemoattractant protein-1; **CBN**: cannabinol; **THC**: tetrahydrocannabinol; **IL-12:** interleukin-12; **CBC**: cannabichromene; **CBG**: cannabigerol; **ACBG:** cannabigerolic acid **CMC:** mean colony growth; **CBD-MW:** CBD-mouthwash; **CBG-MW**: CBG mouthwash; **MIC**: minimal inhibitory growth; **CBRs:** cannabinoid receptors; **JE:** junctional epithelium; **LP:** periodontal ligament; **CT:** connective tissue; **OA:** alveolar bone; **SH-SY5Y:** human neuroblastoma cell line; **POLY I:C**: polyinosinic-polycytidylic acid; **NA**: not applicable; **LPH:** human periodontal ligament; **CTLPH:** human periodontal ligament stem cells.

## Data Availability

The data presented in this study are available on request from the corresponding author.
